# A systematic review and meta-analysis of moderate-to-vigorous physical activity levels in secondary school physical education lessons

**DOI:** 10.1186/s12966-017-0504-0

**Published:** 2017-04-24

**Authors:** Jenna L. Hollis, Rachel Sutherland, Amanda J. Williams, Elizabeth Campbell, Nicole Nathan, Luke Wolfenden, Philip J. Morgan, David R. Lubans, Karen Gillham, John Wiggers

**Affiliations:** 1 Hunter New England Population Health, Locked Bag 10, Wallsend, NSW 2287 Australia; 2 School of Medicine and Public Health, University of Newcastle, Callaghan, 2308 Australia; 3 Hunter Medical Research Institute, Lambton, NSW 2305 Australia; 4 Priority Research Centre in Physical Activity and Nutrition, School of Education, University of Newcastle, Callaghan, 2308 Australia

**Keywords:** High school, Middle school, Lesson, Class, PE, Exercise, MVPA, Student, Adolescent

## Abstract

**Background:**

Schools play an important role in physical activity promotion for adolescents. The systematic review aimed to determine the proportion of secondary (middle and high) school physical education (PE) lesson time that students spend in moderate to vigorous physical activity (MVPA), and to assess if MVPA was moderated by school level (middle and high school), type of physical activity measurement and type of PE activities.

**Methods:**

A systematic search of nine electronic databases was conducted (PROSPERO2014:CRD42014009649). Studies were eligible if they were published between 2005 and 2014; written in English; assessed MVPA in PE lessons of secondary (middle and high) school students; and used a quantitative MVPA measure (i.e., accelerometry, heart rate monitoring, pedometers or observational measures). Two reviewers examined the retrieved articles, assessed risk of bias, and performed data extraction. Random effects meta-analysis was used to calculate a pooled estimate of the percent of PE lesson time spent in MVPA and to assess moderator effects where data allowed.

**Results:**

The search yielded 5,132 potentially relevant articles; 28 articles representing 25 studies (7 middle and 18 high school) from seven countries were included. Twelve studies measured MVPA through observational measures, seven used accelerometers, five used heart rate monitors and four used pedometers (including three studies using a mix of measures). Meta-analysis of 15 studies found that overall, students spent a mean (95% CI) of 40.5% (34.8–46.2%) of PE in MVPA. Middle school students spent 48.6% (41.3–55.9%) of the lesson in MVPA (*n* = 5 studies) and high school students 35.9% (28.3–43.6%) (*n* = 10 studies). Studies measuring MVPA using accelerometers (*n* = 5) showed that students spent 34.7% (25.1–44.4%) of the lesson in MVPA, while 44.4% (38.3–50.5%) was found for lessons assessed via observation (*n* = 9), 43.1% (24.3–61.9%) of the lesson for a heart rate based study, and 35.9% (31.0–40.8%) for a pedometer-measured study.

**Conclusions:**

The proportion of PE spent in MVPA (40.5%) is below the US Centre for Disease Control and Prevention and the UK Associations for Physical Education recommendation of 50%. Findings differed according to the method of MVPA assessment. Additional strategies and intervention research are needed to build more active lesson time in PE.

**Electronic supplementary material:**

The online version of this article (doi:10.1186/s12966-017-0504-0) contains supplementary material, which is available to authorized users.

## Background

Moderate-to-vigorous physical activity (MVPA) during adolescence has been positively associated with a host of physiological and psychological outcomes such as cardiorespiratory fitness [1], reduced metabolic disease risk [2] and better mental health [3, 4]. The World Health Organisation (WHO) recommends children and adolescents aged 5 to 17 years old participate in 60 min of MVPA everyday [5]. Internationally, as few as 20% of adolescents meet this recommendation [6]. Schools play an important role in physical activity promotion for adolescents [7], an age that has been associated with declining physical activity levels [8, 9].

The United States’ (US) Centre for Disease Control (CDC) and Prevention [10] and the United Kingdoms (UK) Associations for Physical Education (AfPE) [11] advise that children (5–17 years old) should participate in MVPA for 50% of PE lesson time to gain appropriate health and academic benefits. The most recent review to examine the proportion of PE lesson time spent in MVPA in secondary schools (i.e., middle and high school) was published in 2005 and reviewed 40 studies [12]. The majority of studies used heart rate monitoring to measure MVPA (*n* = 30), 10 studies used systematic observation such as the System for Observing Fitness Instruction Time (SOFIT), and four used accelerometry [12]. When data from the studies were combined (not a meta-analysis), the review found that secondary school students engaged in MVPA for between 27% and 47% of PE time depending on the type of measurement instrument used to measure MVPA; accelerometers-assessed MVPA was reported for 46.8 ± 13.9% of lesson time, heart rate monitor-assessed MVPA for 37.9 ± 14.6% and observational-assessed MVPA for 26.6 ± 15.2% of lesson time [12]. Sub-analyses found MVPA levels varied according to the type of activity students engaged in; from almost 50% of lesson time when engaged in fitness orientated activities (48%) or team invasion games (e.g., basketball and soccer; 46%) compared to just one third of time when participating in dance and gymnastics, or net game activities [12]. The review did not examine MVPA in middle and high school PE lessons separately.

Since 2005, numerous observational and intervention studies examining MVPA in middle and high school PE lessons have been conducted. However there has been no systematic review to examine the proportion of secondary school PE lesson time, without intervention, spent in MVPA. While it is important to acknowledge that the target of 50% MVPA is only one aspect of assessing the quality of PE lessons, continued monitoring of guideline implementation is important as a tool to track any improvements in MVPA or achievements of PE lesson targets. Therefore, the primary aim of this systematic review was to update the evidence base and determine the proportion of secondary (middle and high) school PE lesson time that students spend in MVPA. Secondary aims were to evaluate student participation in MVPA during PE lesson time according to three potential moderators, namely: i) school level (middle or high schools); ii) type of physical activity measurement (accelerometer, heart rate monitoring, pedometry or observational measure); and iii) type of PE activities (fitness orientated activities, team invasion games, dance and gymnastics or net game activities).

## Methods

### Search Strategy

The systematic review protocol was registered with Prospero on the 7^th^ May 2014 (http://www.crd.york.ac.uk/PROSPERO/display_record.asp?ID=CRD42014009649; PROSPERO2014:CRD42014009649). The systematic review adhered to the Preferred Reporting Items for Systematic Reviews and Meta-analysis (PRISMA) statement [13]. A two-step search strategy was used. Firstly, a search using key words was carried out across nine electronic databases: Medline, Sport Discus, CINAHL, The Central Cochrane database of Systematic Reviews, CENTRAL, ERIC Proquest, EMBASE, Scopus and PsycINFO. Key search terms and their synonyms were searched separately in four main filters which were then combined. Search filter one identified the setting such as ‘physical education’, ‘lesson*’, ‘class*’. Search filter two referred to the target population including ‘child’, ‘adolesc*’ and ‘student’. Measurement terms were identified using search filter three such as ‘motor activity’, ‘exercise’ and ‘MVPA’. Search filter four identified the study design including ‘prospective studies’, ‘longitudinal studies’, ‘non-randomized’. Search terms within each filter were combined using the Boolean operator ‘or’, and all four filters were combined to form one search using the Boolean operator ‘and’. See Additional file 1 for a record of the search strategy used for each database. In the second step of the search strategy, the reference list of the included studies was manually searched for additional papers not previously identified.

The title, abstract and description/MESH heading of the studies identified during the search were retrieved and examined by two independent reviewers (JLH and RS) to determine if the study met the inclusion criteria. The full texts of the potentially eligible studies were retrieved and independently assessed by the two reviewers for eligibility. If the two independent reviewers disagreed on whether a study should be included in the review, a third independent reviewer (EC) was consulted until a consensus was reached.

### Inclusion and exclusion criteria

This review considered studies i) published in English from 2005 to 2014; ii) that assessed the physical activity levels of students during PE lessons at a secondary school [middle (i.e., Grade 6–8; approximately 10–14 years old) or high school (i.e., Grade 7–12; approximately 12–18 years old)]; iii) included a quantitative measure of physical activity levels (e.g., accelerometry, heart rate monitoring, pedometers or systematic observational measures); iv) were of cross sectional or prospective longitudinal quantitative design, or the baseline intervention and/or control group results of randomised controlled trials (RCTs), non-randomised controlled trials (non-RCTs) and pre-post studies. The control group results during the study period were included if no baseline control data was provided and the control group received no intervention. The review excluded studies that reported on preschool or elementary/primary school children, abstracts, theses/dissertations and unpublished literature, were published prior to 2005, and reported on only the follow-up study results from interventions in RCTs, non-RCTs and pre-post studies.

### Assessment of risk of bias

An 11-item tool was developed to assess the risk of bias of the included studies (Additional file 2). The tool was created as no existing risk of bias tool assessed bias that was relevant to the topic. For example, existing tools assessed studies on participant recall bias, interviewer bias, the randomisation procedure and attrition [14–16]; all criteria which were not directly relevant to this systematic review. The existing tools lacked detailed criterion regarding selection and instrument bias across the school, class and student level, which were more likely to influence the findings. The tool consisted of seven domains covering selection bias at the school, class and student level, plus selection and instrument bias related to the PE lessons and MVPA measures. The tool was used by the authors in a previous systematic review of MVPA in elementary school PE lessons [17]. In this review, two independent reviewers (JLH and RS) used the tool to assess the risk of bias of all studies included in the systematic review. Any disagreements were resolved through discussion between the two reviewers, and if a consensus could not be reached, a third reviewer was consulted (EC). Each of the 11 criteria was coded as ‘clearly described and present’ (yes), ‘absent’ (no), or ‘unclear and/or inadequately described’ (unclear) for each study. Each domain was considered independently as recommended by PRISMA [13].

### Data collection

Data were extracted from the retrieved papers for evidence synthesis using a pre-piloted standardised data extraction table developed by the authors. One independent reviewer (JLH) extracted the data, and a second independent review (RS) examined the completed data extraction table, added any missing information, corrected errors and highlighted any data that were unclear. The two reviewers discussed all discrepancies. A third reviewer (EC) was consulted if a consensus could not be reached. Missing data were requested from study authors if necessary to determine study eligibility and where insufficient data were provided for inclusion in the meta-analysis. The extracted data included study design, the setting (region/country, middle school, high school, school level), participants (school and student sample size, student age, sex, socioeconomic status (SES), ethnicity), teacher training, aim, recruitment, response rate, measurement type, lesson delivery, number of lessons, lesson duration, and activities engaged in during the lesson. MVPA in PE lessons was extracted as either: i) mean percentage of lesson time spent in MVPA, or ii) minutes of MVPA per lesson and length of the PE lesson so that percentage MVPA per lesson could be manually calculated. The activities engaged in during the lesson/s were extracted verbatim from the study description, with the intention to re-categorise these into four categories as in the previous review [12]: i) fitness orientated activities, ii) team invasion games, iii) dance and gymnastics, and iv) net game activities. If lessons contained activities that fell into more than one category, data on the time spent in each activity from the different categories were also extracted (if reported).

### Data synthesis

Data were synthesised via a narrative description of findings from the included studies. Summaries of the physical activity levels in each study were presented as both mean (SD/SE/95%CI) percentage of time and actual minutes, if provided. Comprehensive Meta-analysis Software (version 2.2.064, July 2011) was used to pool the findings into a meta-analysis for studies that reported i) mean percentage of time in MVPA, ii) a standard deviation, and iii) the number of PE lessons observed. Findings were combined for the main meta-analysis regardless of the assessment method. Percentage time spent in MVPA was quantified from pedometer steps per minute by the authors (JLH and RS) using a standardised equation [18]. The meta-analysis was weighted by inverse variance assuming a random-effects model, according to the number of PE lessons monitored in each study. A decision to assign study weighting based on PE lessons was made as the factor variable of interest is the variability of MVPA at the lesson level, not the student level. As studies reported the measure at an aggregate lesson level (e.g., average of 39% of the lessons spent in MVPA) and the student sample size for each individual lesson was not clear for all studies, we are unable to assign study weighting by student sample size. A larger number of PE lessons monitored in a study would provide a more accurate estimate of percentage MVPA in usual PE lessons. Providing that either i) all students in a PE lesson are monitored, or ii) students are randomly selected for monitoring (i.e., the protocol for observational assessment using SOFIT), then the average student percentage MVPA data collected should be representative of percentage MVPA in the assessed lesson regardless of the number of students monitored.

Moderator analyses were performed to determine percent MVPA by school level (middle or high school), type of physical activity measurement (accelerometer, heart rate monitor, pedometer or observational measure) and type of PE activities (fitness orientated activities, team invasion games, dance and gymnastics or net game activities). Cochran’s Q and the *I* ^*2*^ Index tests were used to assess statistical heterogeneity. For the *I* ^*2*^ Index; 0-40% may represent low heterogeneity; 30–60% moderate heterogeneity; 50–90% substantial heterogeneity; and 75–100% considerable heterogeneity [14]. The moderator meta-analysis that examined the method of measuring MVPA was also used to examine methodological heterogeneity.

## Results

### Description of the studies

**Fig. 1:**
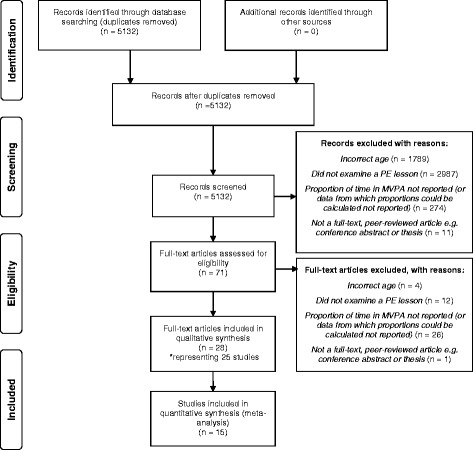
PRISMA flow chart illustrating study inclusions through the stages of the systematic review and meta-analysis

The stages of the systematic review and study exclusions are shown Fig. 1. The initial database search returned 8,300 journal articles prior to de-duplication, or 5,132 journal articles once duplicates were removed. Following title and abstract review, 71 full text articles were retrieved and assessed for eligibility. Twenty-eight papers representing 25 studies (7 middle school and 18 high school studies) met all inclusion criteria and were included in the systematic review. All study selection discrepancies between the two reviewers were resolved through discussion and the third reviewer was not consulted. Missing data were requested from 13 study authors to determine study eligibility and/or to obtain sufficient data for the study to be pooled into the meta-analysis. Nine authors responded to the email communication, of which five provided the additional requested data. No additional eligible articles were retrieved from the reference lists of the included articles.

### Characteristics of included studies

The characteristics and outcomes of the studies are shown in Tables 1 and 2. Publication dates ranged from 2005 [19–23] to 2014 [24, 25]. The studies were primarily conducted in the United States of America (USA; *n* = 13) and Australia (*n* = 6), with two studies in Portugal and one each in the United Kingdom, Poland, Brazil and Hong Kong. All seven middle school studies were conducted in the USA. Almost 50% of the studies were of cross-sectional design (*n* = 12), followed by RCT’s (*n* = 4), non-RCT’s (*n* = 3), pre-post studies (*n* = 2), quasi-experimental RCT (*n* = 1), cluster RCT (*n* = 1), single subject multi-element study (*n* = 1) and a group randomised serial cross-sectional study (*n* = 1). Of the 12 studies that were not of cross-sectional nature, six studies contributed baseline intervention and/or control group data to the systematic review [19, 20, 26–31] and the remaining six studies contributed only control group data at follow-up [21, 23, 32–35].

**Table 1:** Methodological characteristics of secondary school PE lesson studies included in the systematic review

Reference: first author, year & study type	Sample size, schools (n), students (n)	Population age (mean (SD) or range), sex (% female), SES, ethnicity, geographic location	Aim, study groups included in analysis	Recruitment method, response rate (%)	Outcome measures	Measure of MVPA	PE lesson delivery, season/month & year	Number of PE classes observed, minutes	PE activities
Middle Schools									
Barroso et al. 2009 [36] Cross-sectional study	Schools: 17 middle schools Students: 6^th^ – 8^th^ grade, number of students NR	Age, sex, ethnicity, SES NR Texas-Mexico border, USA	To assess awareness/adherence to Senate Bill 42, & to assess the impact of Senate Bill 42 on the frequency/quality of structured PA, & prevalence of child self-reported PA behaviours & child overweight along the Texas-Mexican border ^a^ Group A = subsample of 17 schools on Texas-Mexico border	A subsample (17/112) of 2004/05 SPAN middle schools in 4 Texas-Mexico border metro areas (based on probability sampling). Response rate: NR	Awareness of & adherence to Senate Bill 42, self-report PA & SSR, PE class attendance, MPA, VPA, height, weight, BMI, behavioural characteristics	SOFIT	PE instructors, Female & Male Spring 2007/08	≤3 PE lessons observed per school (1 observation per 6^th^, 7^th^ & 8^th^ grade); classes could be mixed; total = 46	NR (mainly indoor activities)
Coe et al. 2006. [32] RCT	School: 1 public school Student: 214 6^th^ grade students	11.5 ± 0.4 years (range 10–12.8 years); 51% boys, 49% girls; 68% White, 10–14% Hispanic, 3–4% Black, 3-6% Asian, 12% other ethnicity. SES NR Western Michigan, USA	To determine the effect of PE class enrolment & physical activity on academic achievement in middle school children Group A = all PE lessons (no control)	Recruitment: students recruited within school, consent forms sent to parents on first day of school Response rate = 36.8% students (229/622). 93.4% (214/229) completed data collection	Academic achievement, %MPA & %VPA in PE lesson, habitual PA/day, height, weight, BMI	SOFIT	2 PE teachers in the school	8 lessons observed (each teacher (2) observed 4 times throughout the year), 55 min classes/day	NR
Fu et al. 2013 [27] Non-randomised trial	School: 1 urban middle school, 7^th^–8^th^ grade Students: 61 students	12.6 (0.6) years; 41% male. Ethnicity & SES NR Mountain West region, USA	To examine the effects of a health-related physical fitness-based basketball program on middle school students’ in-class physical activity, perceived competence, & enjoyment as compared to the effects on those study variables shown by a control group participating in the traditional approach basketball unit Group A = baseline results of Health related physical fitness basketball unit; Group B = Baseline results of Traditional approach basketball unit	Recruitment: students recruited within school, parents/guardians provided informed written consent Response rate: NR	In-class PA, perceived competence, children’s enjoyment	Pedometer	PE teacher, master’s degree in PE, 30 min training on study protocol (rationale, hands on experience of implementing strategies)	1 × 50 min PE session class/week for 6 weeks (only baseline data included in this analysis)	Basketball. Included aerobic & static/dynamic warm-up (5 min), skill related drills (15 min), game-play (30 min)
Gao et al. 2011 [37] Cross-sectional Study	School: 1 suburban public school, 6-8^th^ grade Students: 149 students, 10–14 years old	12.48 (1.02) years; 50% male; 19.81 kg/m^2^; 22% overweight/obese; 89.3% Caucasian, 7.4% African American, 2% Asian American, 1.3% Hispanic American; mostly middle to high SES families; 28.9% from 6^th^ grade, 36.2% from 7^th^ grade, 34.9% from 8^th^ grade Southern USA	To identify the percentages of students are overweight & obese based on BMI; students’ PA levels in PE as measured by accelerometers; to determine if there are significant differences in students’ PA levels across different BMI groups (healthy weight vs overweight/obese) Group A = all PE lessons (total); then results by weight status (healthy weight & overweight/obese)	Recruitment: recruited within school, permission obtained from participants & parents/guardians Response rate: NR	BMI, MVPA in PE class	Accelerometer	3 PE lessons, Taught by PE teachers, teaching on alternate days	2 or 3 ×90 min PE classes/week	Catch-ball, walking/jogging, line dancing, soccer, table tennis. PE time included warm-up routines, activities & games. All classes ended with a lesson assessment
Liu et al. 2013 [34] Non-randomised trial	Schools: two middle schools, 6^th^ grade Student: 247 students; PE4Life = 154 (80 boys, 74 girls), Traditional = 93 (37 boys, 56 girls)	11.58 ± 0.61 & 11.40 ± 0.46 years (mean age of 2 groups); 47.4% male; median income of groups ~ $30,000; >95% white ethnicity Region NR, USA	To compare health-related physical fitness of 2 samples of 6^th^ grade student enrolled in 2 different PE programs (PE4life Program & a traditional PE program), which were in striking contrast in their MVPA levels in PE class Group A = traditional PE program	Recruitment: all 6^th^ graders from 2 schools invited to participate Response rate: NR	MVPA in PE lessons, % body fat, BMI, progressive aerobic cardiovascular endurance run, fitness test	SOFIT	PE specialists, 9 years of experience, aged 36–52 years old	43 lessons observed at each school, 90 min PE/week	PE4life Program: Individual/lifetime sports & strength/fitness activities Traditional PE program: competitive team sports & other PA
McKenzie et al. 2006 [29] RCT	Schools: 36 public schools, 6 field sites Students: mean (SD) 1027 (285) students/school	100% girls; 47% non-white. 34% received free or low cost meals. Age & SES NR. Multi-site, USA	To assess girls’ PA in middle school PE as it related to field site, lesson context & location, teacher gender, & class composition Group A = baseline value from the TAAG study schools	Recruitment: Schools participating in TAAG Response rate: NR	MVPA in PE lessons, lesson context, activity promotion	SOFIT	60% taught by female PE teachers Jan-May 2003	431 lessons (70–74 per site). Mean lesson length 37.3 ± 9.4 min. 30 students/class 83% in co-educational format. Observations occurred over 3 days/school	NR 65% held indoors
Springer et al. 2013 [38] Group randomized Serial Cross-sectional Study	School: 30 public middle schools, 6^th^-8^th^ grade (MVPA collected in subsample of 21 schools) Students: NR, only 6^th^ - 7^th^ grade students provided data on MVPA in PE lessons	Whole sample (6^th^-8^th^ grade) demographics - 13.9 years; 51.2% female; 37.9% overweight & 19.2% obese; 51.9% Hispanic, 25.1% White, 13.2% African American; 58.7% economically disadvantaged Texas, USA	The 3.5 year CATCH Middle School Project aimed to promote PA, healthy eating, & obesity prevention among year 6–8 middle school students & their families. The evaluation aimed to observe changes in energy balance behaviours based on exposure to CATCH over 3.5 years Group A = baseline data from a subsample of 21 schools	Recruitment: 30/32 eligible public schools selected from 5 central Texas independent school districts. Students recruited through verbal & written invitation in core classes. Student assent & parent passive/active consent. Response rate: 100% (30/30) schools agreed to participate, 72% students (2,841/3,944) participated at baseline	BMI, 7-day PA, sedentary behaviour, MVPA in PE lessons, dietary behaviours, related psychosocial constructs including social support, home availability & accessibility of fruit & vegetables	SOFIT	PE teachers	84 observations from 21 schools, 4 randomly selected PE classes (2 × 6^th^ grade & 2 × 7^th^ grade classes)	NR
High Schools									
Bronikowski et al. 2005 [23] Non-randomised trial	School: 1 junior school Students: NR, 4 children measured per PE class	13 years. Sex, ethnicity & SES NR Poznan, Poland	To analyse & present differences between the 2 Polish systems of physical education (3 PE classes/week & 4 PE classes/week) Group A = 3 PE classes/week; Group B = 4 PE classes/week	Recruitment: students recruited within school, parents provided informed consent Response rate: NR	Health related fitness (volume & intensity of PA in PE classes)	HR monitoring	Two PE class schedules of 3 & 4 lessons of PE per week, 45 min each 2002–04	First half of 2002/03 4 PE classes per week (71 classes) Second half of 2003/04 3 PE classes per week (78 classes); separate classes for boys & girls	Athletics, basketball, volleyball, gymnastics, gymnastics jumps, table tennis, motor fitness tests, aerobic, football, other team sports (e.g., floor ball & soft ball)
Chow et al. 2009 [45] Cross-sectional Study	Schools: 30 schools (6% of the countries co-educational school, 10% of boys only & 10% of girls only schools) Students: NR. Class size 5–55, mean 32.8 (9.01)	63 classes in 7^th^ grade 49 classes in 8^th^ grade 62 classes in 9^th^ grade 42 classes in 10^th^ grade 6 classes in 11^th^ grade 16 classes in 12^th^ grade Class size = 32.8 (9.01) students. Age, Sex, SES & ethnicity NR Hong Kong Island, Kowloon, New Territories, Hong Kong	To measure student physical activity, lesson context, & teacher behaviour during physical education lessons in a representative sample of secondary schools in Hong Kong, & assess the influence of class gender composition & other environmental factors (e.g., lesson location, activity areas size, class size) on students’ physical activity levels during those lessons Group A = all PE lessons (no intervention)	Recruitment: NR Response rate: NR	Student PA, lesson context, teacher behaviour	SOFIT	65 PE specialists (38 men, 27 women), mean (SD) age 34.4 (8.5) years old, teaching experience 12 (8.4) years December 2005-May 2006	238 observations each from 123 classes (mean time 57.1 min, range 19–100), randomly selected days	Team activities/sports (basketball, soccer, volleyball, team handball, rugby) individual activities/sports (gymnastics, badminton), expressive activities (dance, rope skipping), other content (fitness training, physical fitness), free play activities
Conley et al. 2011 [26] Pre-post intervention trail	School: 2 co-educational Year 7 PE classes Students: 37 participants	12.6 ± 0.4 years; 40.5% males. SES & ethnicity NR Victoria, Australia	To explore whether children can identify time spent in MVPA, & investigate whether heart rate biofeedback would improve children’s ability to estimate time spent in MVPA Group A = results from 1 pre-intervention lesson	Recruitment: students recruited within school, children & their legal guardians provided written informed consent Response rate: NR	Heart rate measured minutes MVPA, height weight, self-estimated time in MVPA	HR monitoring	PE teachers, responsible for lesson planning & delivery, lesson & teaching variation controlled by combing 2 PE classes for the study Term II (Apr-Jun) during schools recreational fitness PE unit	1 × 70- min pre-intervention PE lesson (~55 min practical PE) included in this analysis; Overall 8 PE lessons monitored over 7 weeks (3 lesson scheduled per 2 weeks)	Circuit lesson: 10 min warm up game, circuit activity stations (running, jumping, hopping, stepping)
Dudley et al. 2012a [39] & Dudley et al. 2012b [40] Baseline cross-sectional study/Longitudinal study	School: 6 schools (2 co-educational, 2 boys-only, 2 girls-only), Year 7 PE classes at baseline, Year 8 PE classes at 1 year follow-up Student: NR, 4 children per class (*n* = 81 lessons at baseline; 51 at follow-up) randomly selected to be observed	12.8 (0.5) years; 55% girls; 38% spoke English as main language at home; 60% resided in suburbs in the 5 deciles of greatest socioeconomic disadvantage. Ethnicity NR South-West Sydney, NSW, Australia	*Dudley* et al. *2012a -* To determine the levels of PA, lesson context & teacher interaction students receive during PE in secondary schools in NSW, Australia *Dudley* et al. *2012b -* To examine the percentage of class time spent participating in PA, lesson context & teacher interaction during secondary school PE & how those variables changed over time from Year 7 to Year 8 Group A = baseline Year 7 results; Group B = follow-up Year 8 results	Recruitment: Collected from PALDC project. School identified by NSW Department of Education & Communities (high & linguistically diverse backgrounds). All identified schools/Year 7 students invited to participate (follow-up in Year 8) Response rate: >99%, 658 students consented to demographic data taken; 504 (77%) at follow-up	PA levels, lesson instruction content, school type	SOFIT	All PE teachers at the 6 schools, PE teachers given <1 week noticed of PE lesson to be monitored, mean of 24 students/class Baseline Jul-Dec 2008; Follow-up Jul-Dec 2009	81 PE lessons monitored at baseline over 5 months (3 randomly selected PE lessons on 3 separate days for each class at each school), 51 PE lessons monitored at follow-up	NR
Fairclough et al. 2005 [12, 19]& Fairclough et al. 2006 [20] ^b^ Quasi-experimental RCT	School: 1 c-educational high schools, 2 Year 7 girls PE classes (30 students each) Students: 33 girls consented (11–12 years old), data analysed on 26 girls, 4 girls/class monitored with SOFIT	~12.4 ± 0.3 years; 100% girls. ethnicity & SES NR Merseyside (North-West England), England	*Fairclough* et al. *2005* - To examine whether a teaching intervention could enhance girls’ physical education levels. To assess whether the intervention compromised the attainment of planned lesson objectives, & levels of intrinsic motivation & perceived competence *Fairclough* et al. *2006* - To increase cardio respiratory health-enhancing physical activity levels during girls’ gymnastics lessons by manipulating the lesson contexts & teacher behaviours, & to achieve this without compromising other planned lesson objectives Group A = baseline intervention data; Group B = baseline control data	Recruitment: students recruited within school, informed written consent provided Response rate: 55% of students (33/60)	Anthropometric, PA levels in PE, psychological characteristics (intrinsic motivation & perceived competence), teacher evaluations	SOFIT & HR monitoring	1 male & 1 female specialist physical education teachers, > 4 years teaching experience, teachers took usual class	1 × 2 h period/week, classes taught in mixed-ability, single sex groups (30–32 students per class). Actual lesson length 82.4 min (control) & 76.0 min (intervention). 6 unit lesson observed in the intervention & control groups. Only 1 baseline lesson for intervention & control groups included in this analysis	Gymnastic lessons
Ferriera et al. 2014 [24] Cross-sectional study	Schools: 3 Portuguese public schools Student: 191 students (12–17 years old)	14.55 ± 1.79 years; 51% male. SES & ethnicity NR Castelo Branco district, Portugal	To determine the amount of MVPA undertaken during a PE class by using an accelerometer, & to verify if the recorded values are in line with the recommended guidelines Group A = all PE lessons (no intervention)	Recruitment: students recruited within school, informed written consent provided by parent/guardian & the school director Response rate: NR	PE class MVPA according to age & gender	Accelerometer	Specialised physical activity educator 2007/08	1 × 90 min PE class/week examined for each school, 6 × 10 min exercises + 30 min instruction/organisation & bathing/changing clothes	Team ball sports (football, handball & basketball), held in outdoor space
Hannon et al. 2005 [21] Pre-post cross over trial	School: 1 Coeducational middle school, 2 PE classes, 9^th^ & 10^th^ grade Student: 78 students	47% male; predominately Caucasian & middle class; age NR North Florida, USA	To compare activity levels, as measured by pedometer step counts per minute, of high school males & females participating in coeducational & single gender flag football game play, & investigate high school girls’ views of participation in co-educational & single gender flag football play Group A = coeducational lesson; Group B = single gender lesson	Recruitment: students recruited within school, parents provided informed written consent Response rate: 100%	PA in PE class	Pedometer	2 PE teachers. 1 × male with 8 years of experience, 1 × female with 14 years of experience	~45 min classes, Single gender & coeducational classes	Flag play (incl. warm up exercises, 10 min skill drills, 20–25 min game). Pedometer only worn for 20 min game
How et al. 2013 [33] RCT	School: 1 independent school, 8 × Year 8 PE classes (4 male classes, 4 female classes) Students: 257	12.91 (0.29) years. Sex, ethnicity & SES NR Western Australia, Australia	To examine whether students within an intervention group, who were provided with choice within PE, reported greater autonomous motivation, more favourable perceptions of autonomy support, & displayed higher in-class PA level than those within a control condition Group A = Regular PE control group	Recruitment: letter sent to parents with passive consent form to withdraw consent. Students provided consent before the study began Response rate: NR	PA levels, PE motivation, autonomy supportive lessons	Accelerometer	4 PE teachers, attended 40 min briefing before study	60 min PE time allocated, ~40 min PA for each lesson	Netball, tennis & tee-ball
Kremer et al. 2012 [41] Cross-sectional study	Schools: 8 secondary schools (16 schools total) Students:84 secondary school students (272 students total), 4 students from each class (2 male, 2 female) randomly selected to be observed during 3 classes	Demographics from primary & secondary schools - 14.3 (2.8) years; 50.2% female; 72.6% white skin colour. SES NR City of Pelotas, Southern Brazil (South of the state Rio Grande do Sul)	To evaluate the intensity & duration of physical efforts in PE classes in primary & secondary school Group A = 1^st^ secondary school; Group B = 2^nd^ secondary school; Group C = 3^rd^ secondary school	Recruitment: list of city’s school obtained, 11 primary & 8 secondary schools drawn, stratified by teaching network (3 primary & secondary schools coincided) Response rate: 100% of schools. Students response rate NR	BMI, MVPA in PE lessons	Accelerometer	NR Measured Aug-Dec 2009	218 classes total, number of secondary school lessons observed NR	NR
Lonsdale et al. 2013 [28, 54] & Rosenkranz et al. 2012 [30] Cluster RCT	Schools: 5 schools (2 independent & 3 Catholic schools), 8^th^ grade, 3 schools provided 4 classes, 2 schools provided 2 classes Students: 288 total	13.6 years; 50.4% male. SES & ethnicity NR Sydney, Australia	To examine the effects of 3 SDT-based motivational strategies on PA & sedentary behaviour, as well as their hypothesized antecedents during PE lessons Group A = baseline data for control/usual practice; Group B = baseline data for intervention ‘relevance’; Groups C = baseline data for intervention ‘providing choice’; Group D = baseline data for intervention ‘free choice’	Recruitment: 20 schools incited to join (9 declined due to time constraints, 5 didn’t respond, 1 was unable to participate due to PE teacher injury). All principals, PE teachers& parents provided written consent Response rate: 80.67% completed baseline assessment, 85.01% completed follow-up (245/288)	PA, MVPA, & student motivation during PE class, sedentary behaviour, perceptions of teacher support, psychological needs satisfactions	Accelerometer	16 PE teachers Oct-Dec 2013	Differing duration of PE lesson, PA data collected in first 20 min of lesson	Dance, netball, touch rugby
Owen et al. 2013 [44] Cross-sectional study	School: 1 independent Catholic boys school, Year 9 students Students: 131 participants, complete data in 61 students	14.36 (0.48) years; 100% male. SES & ethnicity NR Sydney, Australia	To investigate how much of the observed variation in adolescent boys MVPA levels (during PE & leisure time) was explained by individual- & class-level motivation Group A = all PE lessons (no intervention)	Recruitment: students recruited within school, parents provided informed written consent Response rate: 131/180 students enrolled (72.8%), 61/131 students provided complete data (46.6%)	MVPA in PE lessons, motivation towards PE in lessons, motivation towards PA in leisure time	Accelerometer	NR	NR	NR
Sanders et al. 2014 [25] Cross-sectional study	School: 1 Catholic boys schools, 6 PE classes, Year 9 Students: 133 students, analysis on 74 students	14.36 (0.48) years; 22.36 kg/m^2^; 100% male. SES & ethnicity NR Sydney, Australia	To compare i) adolescent boys’ PA bout length in 2 PA contexts; leisure time & PE lessons, & ii) the effect of varying accelerometer epoch length on estimates of MVPA, VPA, MPA, LPA & sedentary behaviour in both contexts Group A = all PE lessons (no intervention)	Recruitment: students recruited within school, students provided voluntary written assent Response rate: 74% of participants consented (133/180), analysis on 74/133 (56%)	MVPA, VPA, MPA, LPA & sedentary behaviour during PE lessons & leisure time	Accelerometer	Regular school PE teacher	12 PE lessons (2 lessons per class × 6 classes)	Soccer
Scruggs et al. 2010a [46] Cross-sectional study	School: 3 high schools Students: 189 students	16.74 (0.99) years; 43.9% male; 169.11 ± 9.10 cm; 67.08 ± 13.02 kg/m^2^; 8.65% obese; 16.76% overweight. SES & ethnicity NR Upper mid-western USA	To compare the relative & absolute agreement between W4L DUO & Yamax SW651 pedometers on the measure of steps/min & the W4L DUO & observed PA time (min) in high school PE Group A = YAMAX SW651 Pedometer used to measured MVPA; Group B = W4L DUO pedometer used to measure MVPA; Group C = SOFIT used to measure MVPA	Recruitment: within the schools (<18 year old students provided parental consent, >18 year old students provided personal consent) Response rate: NR	Steps/min, physical activity time	Pedometer & SOFIT	3 certified PE practitioners representing urban, suburban & rural communities	12 PE classes collected during 16 lessons. Both pedometer measures & SOFIT recorded on the same lesson. Traditional 50 min scheduled lesson (36.72 (4.42) min); Block 90 min scheduled lesson (76.19 (4.17) min)	NR
Scruggs et al. 2010b [47] Cross-sectional study	Schools: 6 high schools (5 public, 1 private), 27 PE classes, 9^th^-12^th^ grade Students: 218 students (16 students/class wore a pedometer)	16.52 (1.08) years; 169.61 (9.23) cm; 67.44 (12.69) kg; 23.41 (3.99) kg/m^2^; 49.5% male; 15% non-Caucasian. SES NR Upper mid-western USA	To quantify the recommended minimum level (i.e., 50% of the class time) of MVPA within high school PE via pedometry/min, and to explore the influence of lesson duration (i.e., traditional v’s block schedules) on quantifying MVPA via steps/min Group A = all PE lessons (no intervention)	Recruitment: within the schools (<18 year old students provided parental consent, >18 year old students provided personal consent) Response rate: NR	Steps/min, % time engaged in MVPA, time engaged in MVPA	Pedometer	10 certified physical educators	27 PE classes (traditional class 45–50 min; block class 90 min), 40 PE lessons (30 traditional; 10 block). Traditional lesson 36.88 (4.07) min; block lesson 78.56 (5.08) min	Block lessons: dance, invasion game & fitness course themes. Traditional lessons: Fielding, invasion & net wall games, dance/gymnastics, fitness course, ropes/team building themes
Surapiboonchai et al. 2012 [43] Cross-sectional study (validation study)	School: 6 schools, grades 3,5,6,7,8,9,10 (only grade 6–10 examined in this review) Students: 281 students total (all grades); HR = 36 (24 students from middle & high school); SAM = 281 (high & middle school students NR)	Grade 6: 12.33 (1.16) years, 20.0 (5.66) kg/m^2^ Grade 7: 12.00 (0.01) years, 34.67 (4.51) kg/m^2^ Grade 8: 13.86 (0.90) years, 24.40 (4.04) kg/m^2^ Grade 9: 14.13 (0.35) years, 35.17 (7.89) kg/m^2^ Grade 10: 15.00 (0.01) years, 40.50 (3.25) kg/m^2^ Whole student sample demographics - 50% male; 92% economically disadvantaged’ 89.5% Hispanic, 7.4% African American, 2.7% White San Antonio, Texas, USA	To develop, validate & test the reliability of the Simple Activity Measurement (SAM) instrument for assessing student MVPA during school PE classes related to the potential for evaluating the achievement of ≥ 50% of PE class time spent in MVPA Group A = all PE lessons (no intervention)	Recruitment: parent or student consent not required as this area was required as a part of general PE curriculum. Response rate: NR	MVPA in PE lessons	SAM Tool (observational tool)	PE teachers late fall 2009	6 PE classes observed with SAM tool; 45–50 min lessons (17–62 students per class)	Variety of PE units including basketball, handball & fitness conditioning
Vidoni et al. 2012 [31] Single subject multi-element study	School:1 Kindergarten – year 12 public school, 8^th^ grade only assessed, 1 PE class Students: 18 students	13 -14 years old; 55.6% male; middle class SES. Ethnicity NR Midwestern, USA	To investigate the effects of a group dependent contingency strategy called Fair Play Game on students’ heart rates in PE lessons Group A = Baseline results only (4 days of lessons)	Recruitment: within school, parental, teacher & student consent obtained Response rate: NR	MVPA in PE lessons, heart rate, social validity	HR monitoring	Male PE teacher, 20 years teaching experience, 14 years teaching & coaching basketball at study school	Every lesson for 15 days, 35 min lesson (5 min warm-up, 15 min practice drills, 12 min game, 3 min closure) Only baseline (4 days of lessons) results included	Basketball
Wang et al. 2005 [22] Cross-sectional study	School: 1 school, 7^th^ grade, co-educational PE classes Students: 28 students	12.5 years (boys) & 12.1 years (girls); 50% male; 1.51–17.6 cm; 40–80 kg; 17.1–28.9 kg/m^2^. SES & ethnicity NR Northern Portugal	To use a new heart rate monitor to investigate Portuguese 7^th^ grade students’ PA levels during the different indoor PE classes Group A = 90 min lesson; Group B = 45 min lesson	Recruitment: School recruitment NR. Students randomly selected from a total sample of 264 students Response rate: NR	PA levels during indoor PE classes	HR monitoring	PE educators, 25–45 years old	14 indoor PE classes (7 × 45 min & 7 × 90 min classes)	Football, basketball, handball, volleyball, gymnastics, & skill evaluation (all indoor setting 900 m^2^)
Young et al. 2006 [35] RCT	School: 1 all-girls public high school, 9^th^ grade Student: 221 girls	13.8 ± 0.5 years; 100% female; 83.0% African American, 56.3% of girls’ mothers had a high school education. SES NR Baltimore Magnet High School, USA	To test the effectiveness of a life skills-orientated PA intervention, conducted in PE class by a teacher hired by the project, for increasing PA & fitness in 9^th^ grade girls Group A = standard PE class (control).	Recruitment: parent & student orientation meetings, mass mailings to parents, classroom presentations to student. Students recruited over 3 successive years. Informed consent from parent/legal guardian. Response rate: 50% (221/442), 95% retention	Self-report daily PA, self-report sedentary activities, cardiorespiratory fitness, CVD risk factors (e.g., BMI, waist circumference, blood pressure)	SOFIT (modified version)	Certified PE teachers at the school (control only) Baseline measures in Sept	81 total PE classes (41 control), 45 min class lesson	Individual & team sports (e.g., basketball). Specific sports NR

*PA* physical activity, *SOFIT* System for Observing Fitness Instruction Time, *HR* Heart rate, *yrs* years, *MVPA* moderate-to-vigorous physical activity, *PE* physical education, *NR* not reported, *NSW* New South Wales, *SSR* Small Screen Recreation, *PALDC* Physically Active in Linguistically Diverse Communities, *BMI* Body Mass Index, *incl*. including, *SDT* Self-Determination Theory, *TAAG* Trial of Activity for Adolescent Girls, *VPA* vigorous physical activity, *MPA* moderate physical activity, *LPA* light physical activity, *min* minutes, *RCT* Randomised Controlled Trial, *CVD* cardiovascular disease, e.g., for example. i.e., that is

^a^Senate Bill 42 required middle-school children in Texas to participate in 30 min MVPA/day or a minimum of 135 min/week or 225 min/fortnight. Children must also participate in PE for 4/6 semester middle school cycles

^b^Was not pooled into a meta-analysis as only one lesson observed and hence no mean (SD) is reported

**Table 2:** Summary table of the findings of the secondary school PE lesson studies included in the systematic review

Reference: first author & year		Measure	Group A				Group B				Group C				Group D			
			Lessons (n)	MVPA, mean mins ± SE/(SD)	Mins obs, mean ± SE/(SD)	% MVPA, mean ± SE/(SD)	Lessons (n)	MVPA, mean mins ± SE/(SD)	Mins obs, mean ± SE/(SD)	% MVPA, mean ± SE/(SD)	Lessons (n)	MVPA, mean mins ± SE/(SD)	Mins obs, mean ± SE/(SD)	% MVPA, mean ± SE/(SD)	Lessons (n)	MVPA, mean mins ± SE/(SD)	Mins obs, mean ± SE/(SD)	% MVPA, mean ± SE/(SD)
Middle Schools																		
Barroso et al. 2009 [36]		SOFIT	46	NR	51.3 (NR)	54.9 ± 5.1	–	–	–	–	–	–	–	–	–	–	–	–
Coe et al. 2006. [32]		SOFIT	NR	19 (NR)	~55 (NR)	~34.5 (NR)	–	–	–	–	–	–	–	–	–	–	–	–
Fu et al. 2013 [27]		Ped	1	70.35 (12.82) steps/min	~50 (NR)	<50% (NR)	1	61.28 (11.84) steps/min	~50 (NR)	<50% (NR)	–	–	–	–	–	–	–	–
Gao et al. 2011 [37]	Total	Accel	3	~59.96 (NR)	~90 (NR)	66.62 (17.23)	–	–	–	–	–	–	–	–	–	–	–	–
	Healthy weight		3	~61.35 (NR)	~90 (NR)	68.17 (15.56)	–	–	–	–	–	–	–	–	–	–	–	–
	Overweight/obese		3	~55.03 (NR)	~90 (NR)	61.14 (21.54)	–	–	–	–	–	–	–	–	–	–	–	–
Liu et al. 2013 [34]		SOFIT	43	NR	NR	46.10 (9.77)	–	–	–	–	–	–	–	–	–	–	–	–
McKenzie et al. 2006 [29]		SOFIT	431	13.9 (7.0)	37.3 (9.4)	37.9 (18.5)	–	–	–	–	–	–	–	–	–	–	–	–
Springer et al. 2013 [38]		SOFIT	84	NR	NR	50.9 (15.0)	–	–	–	–	–	–	–	–	–	–	–	–
Secondary Schools																		
Bronikowski et al. 2005 [23]	Boys	HR	39	~13.9 (NR)	~45 (NR)	36.5 (NR)	38	~25.3 (NR)	~45 (NR)	46.0 (NR)	–	–	–	–	–	–	–	–
	Girls		32	~13.8 (NR)	~45 (NR)	34.4 (NR)	40	~23.3 (NR)	~45 (NR)	40.9 (NR)	–	–	–	–	–	–	–	–
Chow et al. 2009 [45]	Total	SOFIT	238	19.8 (8.9)	57.1 (NR)	34.8 (13.0)	–	–	–	–	–	–	–	–	–	–	–	–
	Boys		92	21.0 (9.7)	57.1 (NR)	38.2 (14.7)	–	–	–	–	–	–	–	–	–	–	–	–
	Girls		108	18.5 (7.8)	57.1 (NR)	31.8 (12.5)	–	–	–	–	–	–	–	–	–	–	–	–
	Co–ed		38	20.4 (9.3)	57.1 (NR)	35.3 (12.5)	–	–	–	–	–	–	–	–	–	–	–	–
Conley et al. 2011 [26]		HR	1	28 (6.4)	~54 (NR)	51.9 (11.9)	–	–	–	–	–	–	–	–	–	–	–	–
Dudley et al. 2012a [39] & Dudley et al. 2012b [40]		SOFIT	81	33.6 (11.1)	59 (NR)	56.9 (18.7)	51	~30.7 (NR)	59 (NR)	52.1 (24.1)	–	–	–	–	–	–	–	–
Fairclough et al. 2005 [12, 19] & Fairclough et al. 2006 [20] ^a^	HR	HR	1	19.6 (NR)	59.1 (NR)	33.2 (7.4) ^a^	1	24.8 (NR)	85 (NR)	29.2 (20.6) ^a^	–	–	–	–	–	–	–	–
	SOFIT	SOFIT	1	~7.6 (NR)	59.1 NR)	12.9 (NR) ^a^	1	~14.9 (NR)	85 (NR)	17.5 (NR) ^a^	–	–	–	–	–	–	–	–
Ferriera et al. 2014 [24]	Total	Accel	1	25.36 (15.69)	90 (NR)	28.18 (NR) ^a^	–	–	–	–	–	–	–	–	–	–	–	–
	Boys	Accel	1	28.95 (16.02)	90 (NR)	32.17 (NR)^a^	–	–	–	–	–	–	–	–	–	–	–	–
	Girls	Accel	1	21.58 (14.48)	90 (NR)	23.98 (NR) ^a^	–	–	–	–	–	–	–	–	–	–	–	–
	12 years	Accel	1	21.58 (14.48)	90 (NR)	23.98 (NR) ^a^	–	–	–	–	–	–	–	–	–	–	–	–
	13 years	Accel	1	30.40 (16.20)	90 (NR)	33.77 (NR) ^a^	–	–	–	–	–	–	–	–	–	–	–	–
	14 years	Accel	1	29.69 (18.92)	90 (NR)	32.99 (NR) ^a^	–	–	–	–	–	–	–	–	–	–	–	–
	15 years	Accel	1	25.82 (16.14)	90 (NR)	28.69 (NR) ^a^	–	–	–	–	–	–	–	–	–	–	–	–
	16 years	Accel	1	23.33 (13.37)	90 (NR)	25.93 (NR) ^a^	–	–	–	–	–	–	–	–	–	–	–	–
	17 years	Accel	1	22.35 (14.39)	90 (NR)	24.83 (NR) ^a^	–	–	–	–	–	–	–	–	–	–	–	–
Hannon et al. 2005 [21]	Boys	Ped	NR	87.1 (10.6) steps/min	~20 (NR)	~50% (NR)	NR	88.8 (9.5) steps/min	~20 (NR)	>50% (NR)	–	–	–	–	–	–	–	–
	Girls		NR	66.2 (6.3) steps/min	~20 (NR)	<50% (NR)	NR	57.9 (8.6) steps/min	~20 (NR)	<50% (NR)	–	–	–	–	–	–	–	–
How et al. 2013 [33]	Total	Accel	32	~8.4 (NR)	~40 (NR)	21.12 (6.22)	–	–	–	–	–	–	–	–	–	–	–	–
	Boys	Accel	16	~9.3 (NR)	~40 (NR)	23.15 (5.68)	–	–	–	–	–	–	–	–	–	–	–	–
	Girls	Accel	16	~7.6 (NR)	~40 (NR)	19.11 (6.12)	–	–	–	–	–	–	–	–	–	–	–	–
Kremer et al. 2012 [41]		Accel	26	~10.29 (NR)	~35 (NR)	29.4 (24.0)	25	~9.94 (NR)	~35 (NR)	28.4 (25.3)	23	~9.56 (NR)	~35 (NR)	27.3 (26.2)	–	–	–	–
Lonsdale et al. 2013 [28, 54]	Base	Accel	16	7.25 ± NR	20 ± NR^@^	36.25 ± 9.54	16	8.14 ± NR	20 ± NR^@^	40.70 ± 9.47	16	7.03 ± NR	20 ± NR^@^	35.15 ± 9.46	16	7.58 ± NR	20 ± NR^@^	37.88 ± 9.46
	F/U		16	7.29 ± NR	20 ± NR^@^	36.47 ± 9.54	–	–	–	–	–	–	–	–	–	–	–	–
Owen et al. 2013 [44]		Accel	1	20.39 (14.10)	60 (NR)	33.99 (11.95)	–	–	–	–	–	–	–	–	–	–	–	–
Sanders et al. 2014 [25]		Accel	12	NR	NR	36.0 (12.3)	–	–	–	–	–	–	–	–	–	–	–	–
Scruggs et al. 2010a [46]	Overall	SOFIT & Ped	16	15.88 (8.27)	54.89 (10.19)	29.27 (13.60)	16	26.50 (13.40)	54.89 (10.19)	46.96 (18.26)	16	15.17 (7.99)	54.89 (10.19)	30.51 (14.82)	–	–	–	–
	Boys		16	18.02 (7.84)	54.89 (10.19)	35.15 (13.86)	16	29.71 (12.50)	54.89 (10.19)	54.25 (16.33)	16	12.33 (7.79)	54.89 (10.19)	37.10 (14.36)	–	–	–	–
	Girls			14.22 (8.25)	54.89 (10.19)	24.72 (11.55)		24.19 (13.62)	54.89 (10.19)	41.70 (17.83)		18.10 (7.14)	54.89 (10.19)	24.12 (12.31)	–	–	–	–
	Traditional		9	10.51 (5.77)	36.72 (4.42)	29.59 (17.33)	9	16.80 (8.57)	36.72 (4.42)	44.92 (23.18)	9	10.73 (5.64)	36.72 (4.42)	31.26 (18.02)	–	–	–	–
	Block		7	22.12 (6.07)	76.19 (4.17)	28.90 (7.24)	7	37.57 (8.38)	76.19 (4.17)	49.30 (9.70)	7	22.44 (5.57)	76.19 (4.17)	29.28 (6.92)	–	–	–	–
Scruggs et al. 2010b [47]	Overall	Ped	40	17.59 (9.97)	49.69 (19.77)	35.88 (15.83)	–	–	–	–	–	–	–	–	–	–	–	–
	Boys		40	21.29 (13.99)	NR	43.01 (13.99)	–	–	–	–	–	–	–	–	–	–	–	–
	Girls		40	13.96 (8.64)	NR	28.88 (14.38)	–	–	–	–	–	–	–	–	–	–	–	–
	Traditional		30	13.34 (6.56)	36.88 (4.07)	36.63 (17.53)	–	–	–	–	–	–	–	–	–	–	–	–
	Block		10	27.17 (10.03)	78.56 (5.08)	34.20 (11.03)	–	–	–	–	–	–	–	–	–	–	–	–
	9^th^–10^th^ grade		26	21.91 (11.61)	NR	39.24 (13.95)	–	–	–	–	–	–	–	–	–	–	–	–
	11^th^–12^th^ grade		27	13.87 (6.28)	NR	32.99 (16.82)	–	–	–	–	–	–	–	–	–	–	–	–
Surapidoonchai et al. 2012 [43]	Middle school	SAM	3	NR	NR	50.0 (30.8)	–	–	–	–	–	–	–	–	–	–	–	–
	High school		2	NR	NR	36.5 (46.0)	–	–	–	–	–	–	–	–	–	–	–	–
Vidoni et al. 2012 [31]		HR	4	NR	~35 (NR)	43.1 (19.17)	–	–	–	–	–	–	–	–	–	–	–	–
Wang et al. 2005 [22]	Total	HR	7	27.9 (23.9)	61.2 (NR)	~45.6 (NR)	14	6.7 (4.0)	29.5 (NR)	~22.7 (NR)	–	–	–	–	–	–	–	–
	Boys		7	29.7 (23.7)	61.2 (NR)	~48.5 (NR)	7	8.3 (4.6)	29.5 (NR)	~28.1 (NR)	–	–	–	–	–	–	–	–
	Girls		7	26.1 (25.8)	61.2 (NR)	~42.6 (NR)	7	5.0 (2.6)	29.5 (NR)	~16.9 (NR)	–	–	–	–	–	–	–	–
Young et al. 2006 [35]	Girls only	SOFIT ^b^	41	~13.7 (NR)	~45 (NR)	30.5 (NR)	–	–	–	–	–	–	–	–	–	–	–	–

*mins* minutes, *obs* observations, *MVPA* Moderate-to-vigorous physical activity, *SE* standard error, *standard deviation* standard deviation, *base* baseline, *F/U* follow-up, *NR* not reported, *HR* heart rate monitoring, *SOFIT* System for Observing Fitness Instruction Time, *Co-ed* Co-educational; *~* estimated value calculated from the study results provided; ^@^ only a proportion of the total PE lesson was observed

^a^Reported total and standard deviation not reported. Was not pooled into a meta-analysis as only one lesson observed

^b^Modified version of SOFIT

The eligible studies were conducted in 88 middle schools and 77 high schools. Six of the seven middle school studies reported data on both male and female students and did not separate results by sex [27, 32, 34, 36–38] and one study reported a female only sample [29]. Six of the 18 high school studies [26, 30, 31, 39–43] reported data on both male and female students and did not separate results by sex. Of the remaining 12 high school studies, two were conducted with a female only sample [19, 20, 35], two with a male only sample [25, 44], and eight examined both male and female students and reported results separately for each sex [21–24, 33, 45–47].

Twelve studies measured MVPA through observational measures (e.g., SOFIT) [19, 20, 29, 32, 34–36, 38–40, 43, 45–47], seven used accelerometers [24, 25, 28, 33, 37, 41, 44], five used heart rate monitors [19, 20, 22, 23, 26, 31] and four used pedometers [21, 27, 46, 47]. The majority of middle school studies assessed physical activity through observational measures (*n* = 5/7). Studies in a high school setting used a range of measurement tools including observation methods (*n* = 7), accelerometry (*n* = 6), heart rate monitoring (*n* = 5), and pedometers (*n* = 3). Three high school studies used two methods of PE lesson monitoring [19, 20, 46, 47].

The number of PE lessons observed in each study ranged from 1–431. In total, more than 609 middle school PE lessons and 837 high school PE lessons were monitored. One middle school study [32] and one high school study [21] did not report number of lessons monitored. Lesson length was highly variable in both middle school (range: 37–90 min/lesson) and high schools (range: 20–90 min/lesson). All middle school studies (*n* = 7/7) and the majority of high school studies (*n* = 15/18) monitored PE lessons that were led by PE teachers, specialists or instructors. The remaining studies did not state who led the PE lesson [23, 41, 44]. The random effects model was used for main and moderator meta-analyses as there was heterogeneity among the studies (main meta-analysis: Q = 455.8, df = 14, *p* < 0.001, I^2^ = 96.9%; moderator analysis by school level: middle school Q = 70.6, df = 4, *p* < 0.001, I^2^ = 94.3%, high school Q = 274.4, df = 9, *p* < 0.001, I^2^ = 96.7%; moderator analysis by type of physical activity measurement: observational methods Q = 183.7, df = 7, *p* < 0.001, I^2^ = 96.2%, accelerometer methods Q = 49.1, df = 4, *p* < 0.001, I^2^ = 91.9%).

### Risk of bias

The risk of bias coding criteria and results of the appraisal are outlined in Table 3. The representativeness of the school, class and student sample were the primary sources of potential bias. Few studies demonstrated that i) the schools examined were representative of other schools (*n* = 5), ii) the classes chosen to be monitored were representative of all classes (*n* = 9), or iii) the students chosen to be monitored were representative of the population (*n* = 12). All studies adequately described the demographic characteristics of the school sample. The majority of studies used an objective measure of physical activity or cited validation studies (*n* = 22) and stated reliability data (*n* = 22).

**Table 3:** Summary of Risk of Bias assessment for the secondary school PE lesson studies included in the systematic review

Ref: first author and year	Schools level		Class level		Student level			PE lesson observation			
	1. Adequately described the demographic characteristics of the school sample	2. School sample was representative	3. Class chosen was representative of all classes	4. Adequately described the demographic characteristics of the class sample	5. Adequately described the eligibility criteria	6. Adequately described the demographic characteristics of the student sample	7. Student sample representative of the population	8. Described the number of PE lessons observed	9. Objective measure of PA or cited validation studies/stated validity data	10. Objective measure of PA or stated reliability data/cited reliability studies	11. Reported the nature of the physical activities observed
Middle school studies											
Barroso et al. 2009 [36]	Y	Y	U	Y	Y	N	U	Y	Y	Y	N
Coe et al. 2006. [32]	Y	Y	Y	N	N	N	Y	Y	N	N	N
Fu et al. 2013 [27]	Y	U	U	Y	Y	Y	U	Y	Y	Y	Y
Gao et al. 2011a [37]	Y	U	U	Y	Y	Y	U	N	Y	Y	Y
Liu et al. 2013 [34]	Y	U	Y	Y	Y	Y	U	N	N	N	N
McKenzie et al. 2006 [29]	Y	U	U	N	N	N	U	Y	Y	Y	N
Springer et al. 2013 [38]	Y	U	Y	Y	Y	Y	Y	Y	Y	Y	N
High school studies											
Bronikowski et al. 2005 [23]	Y	U	U	N	N	N	U	N	Y	Y	Y
Chow et al. 2009 [45]	Y	Y	Y	Y	Y	N	Y	Y	Y	Y	Y
Conley et al. 2011 [26]	Y	U	U	Y	Y	Y	U	Y	Y	Y	Y
Dudley et al. 2012a [39] & Dudley et al. 2012b [40]	Y	N	Y	Y	Y	Y	Y	Y	Y	Y	N
Fairclough et al. 2005 [12, 19] & Fairclough et al. 2006 [20]	Y	U	U	Y	Y	Y	Y	Y	Y	Y	Y
Ferriera et al. 2014 [24]	Y	Y	Y	Y	Y	Y	Y	Y	Y	Y	Y
Hannon et al. 2005 [21]	Y	U	U	Y	Y	N	U	N	Y	Y	Y
How et al. 2013 [33]	Y	U	U	Y	Y	N	N	Y	Y	Y	Y
Kremer et al. 2012 [41]	Y	Y	Y	Y	N	Y	Y	N	Y	Y	N
Lonsdale et al. 2013 [28, 54] & Rosenkranz et al. 2012 [30]	Y	N	U	Y	Y	Y	Y	N	Y	Y	Y
Owen et al. 2013 [44]	Y	U	Y	Y	Y	Y	Y	N	Y	Y	N
Sanders et al. 2014 [25]	Y	U	U	Y	Y	Y	U	Y	Y	Y	Y
Scruggs et al. 2010a [46]	Y	U	U	Y	Y	Y	U	Y	Y	Y	N
Scruggs et al. 2010b [47]	Y	U	U	Y	Y	Y	U	Y	Y	Y	Y
Surapiboonchai et al. 2012 [43]	Y	N	U	Y	Y	Y	Y	N	Y	Y	Y
Vidoni et al. 2012 [31]	Y	U	U	Y	Y	Y	U	N	Y	Y	Y
Wang et al. 2005 [22]	Y	U	U	Y	Y	Y	Y	Y	Y	Y	Y
Young et al. 2006 [35]	Y	U	Y	Y	Y	Y	Y	Y	N	N	U

Each criteria was coded as ‘clearly described and present’ (yes; Y), ‘absent’ (no; N), or ‘unclear or inadequately described’ (unclear; U) rating for each of the 11 items

### MVPA in secondary school PE lessons

**Fig. 2:**
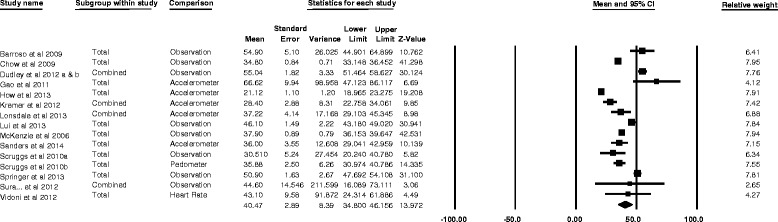
Individual study and pooled results of the percentage of secondary school PE lesson time spent in MVPA

Of the 25 studies included in the systematic review, the percentage of PE lesson time spent in MVPA ranged between 12.9 and 68.2%. Fifteen studies provided the necessary data to be pooled into a meta-analysis. The pooled analysis of these studies (Fig. 2) showed that the mean (95% CI) percentage of PE lesson time that secondary school students spent in MVPA was 40.5% (34.8–46.2%).

### Moderator analyses

**Fig. 3:**
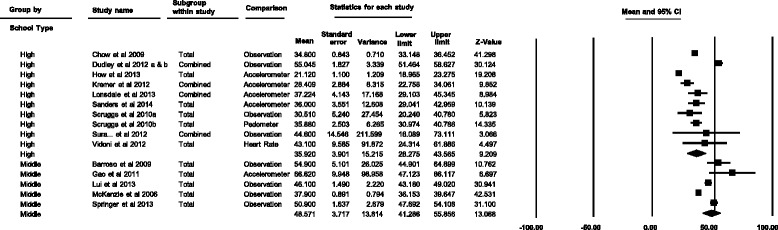
Individual study and pooled results of the percentage of middle and high school PE lesson time spent in MVPA
- *Student participation in MVPA during PE by physical activity measurement type*
  Five of the 15 studies in the meta-analysis assessed MVPA with accelerometers, nine used observational methods, one used heart rate monitors, and one used pedometers. One study used both observational measures and pedometers. For studies using accelerometers, students spent 34.7% (25.1–44.4%) of PE lesson time in MVPA (Fig. 4); in comparison to 44.4% (38.3–50.5%), 43.1% (24.3–61.9%) and 35.9% (31.0–40.8%) when measured using observational methods, heart rate monitors and pedometers, respectively.

- *Student participation in MVPA during PE by school level (middle vs high school)*
  Of the 15 studies included in the meta-analysis, five were conducted in middle schools and 10 in high schools. Middle school students spent a mean (95%CI) of 48.6% (41.3–55.9%) of PE lesson time in MVPA in comparison to 35.9% (28.3–43.6%) of PE lesson time spent in MVPA by high school students (Fig. 3).

**Fig. 4:**
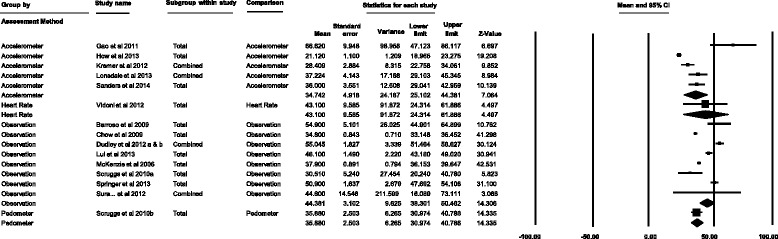
Subgroup meta-analysis of the percentage of secondary school PE lesson time spent in MVPA according to measurement method (accelerometers, heart rate monitors, observational methods and pedometers)
- *Student participation in MVPA during PE by PE lesson type*
  An analysis to examine whether MVPA in lesson time differed by lesson type was not conducted due to a lack of detailed information provided on PE activities. While nine of the 15 studies included in the meta-analysis provided details on the type/s of physical activity that students were engaged in, the majority (7/9 studies) reported on lessons that included activities that fell into multiple categories (fitness orientated activities, team invasion games, dance and gymnastics or net game activities) and the proportion of time spent in MVPA was not reported separately for each lesson type.

## Discussion

### Summary of the evidence

This systematic review and meta-analysis aimed to determine the proportion of secondary (middle and high) school PE lesson time that students spend in MVPA. Of the 25 studies found, the proportion of time spent in MVPA varied considerably between 12.9 and 68.2% of lessons. The meta-analysis of 15 studies found that the proportion of lesson time spent in MVPA is typically below the US CDC [9] and UK AfPE [11] recommendation of 50% of PE lesson time, with only 40.5% of secondary school lesson time spent in MVPA. Middle school students were found to spend a higher proportion of lesson time in MVPA (48.6%) in comparison to high school students (35.9%), although there was considerable variability in the proportions from both groups. Results differed according to the method of MVPA assessment, ranging from 34.7% in accelerometer-assessed lessons to 44.4% for observational-assessed studies. Caution should be taken when interpreting these finding as the confidence intervals for the moderator analyses overlapped and only one study reported findings using heart rate monitors and pedometers. The heterogeneity in study findings may be due to the different ages of the students and the different types of activities in PE (which were often not reported). The studies also differed in their measurement protocols, such as using a variety of measurement instruments (e.g., accelerometers vs SOFIT) with different definitions of reporting time (e.g., scheduled lesson vs actual lesson length), which may have also contributed to the heterogeneity in results between the studies. We were unable to explore differences in MVPA according to lesson type due to a lack of detailed information and data provided on PE activities.

The findings of the overall proportion of MVPA in PE lessons (40.5%) are broadly similar to the previous secondary school review on this topic published in 2005 [12] which reported the proportion of PE lesson time spent in MVPA separately according to the type of measurement instrument used, ranging between 27–47% of the lesson. There were variations in findings between the two reviews according to the type of measurement instrument used. For example, accelerometer-measured lessons reported the lowest levels of MVPA in the present review (34.7%) and the highest proportion MVPA in the previous review (46.8%) [12]. The variation in findings may be explained by differences in methodology between the two reviews; the 2005 review was not a systematic review, did not assess risk of bias and a meta-analysis was not conducted so we are unable to comment on any biases of the component studies which may have influenced the results. The proportion of studies assessed using different measurement instruments also varied between the two reviews, which may have influenced the findings since different instruments may be associated with different types of bias. For example, of the 15 studies included in our meta-analysis, 9 studies used observational measures. Whilst this is a similar number as in the previous review, it forms a higher proportion of the studies (60% v’s 25%). Methodological inconsistencies between the measurement instruments (e.g., length of the monitored PE lesson and the measurement of different elements of activity to calculate MVPA) make it difficult to draw firm conclusions. The implications of methodological inconsistencies and potential biases are discussed in more detail below. Regardless of the type of measurement method used, the findings suggest that overall little progress has been made in engaging adolescents in more MVPA during PE lessons.

Some differences in MVPA levels were observed between studies in the middle and high school setting. The moderator analyses found that, on average, students in middle school PE lessons were observed to be almost meeting the US CDC [9] and UK AfPE [11] recommendation with 48.6% (41.3–55.9%) of lesson time being spent in MVPA, compared with 35.9% (28.3–43.6%) of PE lesson time in high schools. In a recent meta-analysis on MVPA in elementary school PE lessons using the same methodology [17], elementary school students were found to engage in MVPA for 44.8% (28.2–61.4%) of the PE lesson. As the confidence intervals for the elementary, middle and high school analyses overlap, the findings suggest that the level of MVPA in PE across each school setting are relatively comparable. Caution should be taken in interpreting the school level specific results as it is unclear whether the seeming decline in MVPA in PE lessons from middle school to high school was a result of the type of measurement instruments used rather than the school setting. For example, four of the five (80%) middle school studies included in the meta-analysis assessed MVPA using observational methods, in comparison to four of ten (40%) high school studies. The previous review did not examine MVPA in middle and high school PE lessons separately, so we are unable to comment on progress in the different secondary school settings.

As all middle school studies were conducted in the USA, the generalisability of the middle school findings to other countries is unclear. Middle schools in the USA normally enrol students aged between 11–13 years old (6^th^ - 8^th^ grade), although this can vary depending on school districts. This age range spans both elementary and high school ages in other countries such as the UK and Australia. Further research reviewing MVPA in middle school lessons outside of the USA is needed to examine whether the observed level of MVPA in middle school PE lessons is uniform or isolated to the USA. Gaining a better understanding of the strategies implemented to build active lessons in middle school PE lessons in the USA may provide insight to build more active lessons in high schools. Continuing to intervene within the middle school setting remains important for both maintaining activity levels and ensuring activity is undertaken equally across the student population.

Barriers to delivering more active PE lessons have been described as institutional (e.g., school policies, a crowded curriculum, limited facilities and equipment, and insufficient departmental assistance), teacher-related (e.g., related to teachers’ beliefs, skills and confidence) or student-related (e.g., lack of student motivation and interest) [48]. In comparison to elementary schools, fewer teachers-related barriers are reported in secondary school studies where a lack of student motivation and interest emerge as barriers in PE [49]. Evidence suggests that motivation for physical activity engagement may change with maturation [50]. Intrinsic motivation (i.e., enjoyment of the activity) appears to play a leading role in physical activity participation among children, while other forms of autonomous motivations such as identified regulation (i.e., the outcome is identified as personally important), become more important among adolescents [50] and adults [51]. Numerous cross-sectional studies have identified positive associations between controlled and autonomous forms of motivation and physical activity in young people [52], yet empirical studies demonstrating the effect of school-based interventions on student motivation is lacking. There is a clear need for high quality experimental research to evaluate the impact of teacher professional learning interventions on secondary school students’ motivation and MVPA in PE lessons [50] and also to determine any subsequent effects on leisure-time physical activity.

### Risk of bias

The representativeness of the school, class and student sample were the primary sources of potential bias as limited information was provided to determine whether each of these samples were representative of the target population and the studies representative of usual PE lessons. This is not surprising given that many of the studies provided opportunistic data from trials rather than a purposeful sample for PE lesson proportion estimation. As previously noted, all middle school studies were conducted in the USA and the generalisability of findings to other countries for this age group is unclear.

Varied definitions of ‘reporting time’ between different instruments made it challenging to compare studies and may explain some of the differences in findings. Some studies calculated the proportion of MVPA time using the time of the total scheduled PE lesson (e.g., a 60-min lesson) while other studies only monitored physical activity for the time when students are engaged in activity or when a specified proportion of the class are in attendance. For example, accelerometer-assessed lessons typically monitor MVPA for the duration of the scheduled lesson based on school timetables, while the SOFIT observational method monitors student physical activity levels once 51% of students are present, and concludes when 51% of students have left the lesson [53]. Inconsistencies in reporting time, whether stated or not, may distort the results and provide an inaccurate representation of true MVPA time to compare against CDC recommendations [17]. Each instrument also measures different elements of activity to calculate MVPA. For example; accelerometers measure activity using the number of counts above certain cut-points, pedometers according to the number of steps/min, heart rate monitors according to heart rate levels above certain cut-points, and SOFIT uses movement categories. Some forms of activity may be categorised differently depending on the instrument used; for example, SOFIT classifies walking as MVPA while a non-brisk walking pace measured using accelerometers is unlikely to be considered MVPA [17]. Studies that used accelerometers to measure physical activity used different accelerometer cut-points to define MVPA (e.g., >1500 [37], >1962 [24] and >2001 [41] counts/min), which may have also contributed to the variation in findings between studies and made it difficult to compare and summarise the findings.

### Strengths and limitations

This review provides a systematic synthesis of progress in achieving physically active PE lessons. The review included objectively-measured physical activity lessons and also included the addition of pedometer-assessed physical activity in PE lessons. Two moderator analyses were conducted to assess if MVPA differed according to middle or high school PE lesson and type of measurement method. A meta-analysis was conducted to provide an overall pooled estimate of MVPA in secondary schools, as well as for the moderator analyses. The systematic review has some limitations. Seven studies did not provide adequate data to be pooled in to the meta-analysis and, despite several attempts, the study authors were unable to be contacted or were unable to provide the necessary additional information and data. In addition, a further three studies provided data based on one lesson and could not be included in the meta-analysis. As a result, only 15 of the 25 studies were included in the meta-analysis. We did not include any inclusion criteria about probability sampling for any of the study designs. The review was limited to studies that were published in English and in prominent databases. This may not be inclusive of all studies investigating MVPA in PE and published between 2005 and 2014. Studies related to MVPA in PE may appear in a broad range of journals including both educational and health fields, which may not always appear in prominent databases. We were unable to retrieve thesis and conference abstracts.

### Recommendations for future research measuring activity levels in PE lessons

Interventions designed to increase physically active learning time in PE lessons have been recommended as one potential mode of increasing overall MVPA in adolescents, and to promote lifelong activity [54, 55]. Intervention and observational studies investigating physical activity levels in PE lessons remain an important contribution to monitoring progress in the field. We offer the following recommendations to improve the quality of future research:

1. *Standardise the definition of ‘PE lesson time’:* One solution could be reporting MVPA for lessons that monitor within a pre-specified proportion of the lesson (e.g., ≥90%) separately from lessons that monitor a smaller proportion of the scheduled lesson (e.g., <90%) [17]. Or at a minimum, state both the total allocated PE lesson duration and the monitoring time, if different.
2. *Detailed reporting of MVPA outcomes:* Comprehensive reporting is critical to fully monitor progress and maximise the number of studies that are eligible for inclusion in systematic reviews. At minimum, future observation and intervention studies should state the mean MVPA percentage of the lesson, a measure of variation (e.g., standard deviation), minutes of MVPA and the number of lessons examined so that data can be pooled into a meta-analysis.
3. *Report types of physical activities:* Future studies should include a clear description of the activities undertaken, and if possible, provide activity results separately for different types (e.g., fitness orientated, team invasion games, dance and gymnastics and net game activities). This may be challenging if different types of activity are undertaken within one lesson.
4. *Ensure that PE lessons monitored are representative of usual PE lessons:* To increase the representativeness of the findings, studies should monitor lessons from randomly selected schools and classes. All students within the lesson could be assessed, or a random sample of students monitored. Studies should aim to monitor numerous lessons (as many as feasible) as conclusions regarding the proportion of lesson time spent in MVPA can rarely be made from one observation. Information on the representativeness of the sample should also be provided.
5. Transparent and detailed reporting on study information: Many studies were excluded from the review as they lacked important information to confirm eligibility, and study authors were unable to be contacted. Reporting on the lesson context or structure (e.g., time spent in management, knowledge, skill practice, game play and fitness), content (e.g., type of PE activities), delivery (e.g., instructor behaviour) and environment (e.g., where the lesson is delivered and weather) will also enable more comprehensive analysis in future reviews.

### Increasing activity levels in secondary school PE lessons

PE is an opportunity to help students meet the 60 min of MVPA/day recommendation. Maximising MVPA in the existing scheduled PE lesson time could be achieved through targeted strategies such as i) teacher professional learning focused on reducing time spent in class management, instruction and organisation and optimising student motivation, and ii) by supplementing usual PE lessons with high intensity activity such as fitness infusion [54, 56–58]. Incorporating new technologies (e.g., Bluetooth pedometers that sync with apps) that provide real time individualised feedback and summaries of lesson physical activity levels could be tested as a strategy to motivate students and enable teachers to monitor MVPA during PE. Beets et al. [59] have proposed that future youth physical activity promotion interventions be designed according to a framework referred to as the Theory of Expanded, Extended and Enhanced Opportunities (TEO) using one or more of three mechanistic approaches: i) *expansion* – replacing time allocated for low active/sedentary activities with a new occasion to be active, ii) *extension* – increasing the time allocated for physical activity, and/or iii) *enhancement* – modifying existing physical activity opportunities to increase to amount of activity accumulated during an existing period of time. Whatever approach is taken, it is important that PE teachers are supported to balance the need of achieving active PE lessons while also meeting other curriculum and PE educational objectives. Meeting the 50% MVPA target is only one aspect of measuring the quality of PE lessons. Although the MVPA target could be achieved by asking students to run continuous laps of an oval or gym, it is unlikely that this approach will engage students in meaningful learning experiences, and may negatively impact on student’s attitudes, motivation and engagement in physical activity [17, 50, 54]. Not all PE lessons are conducive to high levels of MVPA but may still be, for example, valuable for skill development, fitness, knowledge of movements and improving social and emotional outcomes. For example, a gymnastics lesson may provide an opportunity for students to practice balance and rotation and involve cooperative learning, and may not meet the 50% MVPA target.

## Conclusion

Few studies specifically examining MVPA in PE lessons have been completed, and the studies that have been conducted are difficult to compare due to diverse MVPA measurement methods and incomplete reporting of MVPA outcomes. There is also limited data to comment on the generalisability of study findings. Based on the existing evidence, PE lessons in the secondary school setting fall short of the US CDC and UK AfPE recommendation of 50% of PE lesson time spent in MVPA. Although middle school PE lessons almost meet the MVPA recommendation, intervening in the middle school setting remains important in maintaining activity levels and ensuring that MVPA is undertaken equally across the student population. Further intervention research using the TEO framework [59] is needed to capitalise on building active lesson time in to existing PE lessons, working with schools to develop policies to ensure PE lesson time is protected (i.e., not cancelled) and/or extending the curriculum time allocated to PE. Future studies which monitor PE lessons in secondary schools should aim to incorporate the recommendations made in this review in to the study design to facilitate more accurate and comprehensive monitoring of MVPA in PE lessons.

## Additional file

Additional file 1: A record of the search strategy used for each database. (DOCX 24 kb)
Additional file 2: 11-item risk of bias tool developed for the systematic review. (DOCX 14 kb)

## Abbreviations

95% CI: 95% confidence intervals
AfPE: Associations for physical education
CDC: Centre for disease control
MVPA: Moderate to vigorous physical activity
non-RCTs: Non-randomised controlled trials
PE: Physical education
PRISMA: Preferred reporting items for systematic reviews and meta-analysis
RCTs: Randomised controlled trials
SD: Standard deviation
SE: Standard error
SES: Socioeconomic status
SOFIT: System for observing fitness instruction time
UK: United Kingdom
USA: United States of America
