# Supplementary material for: A systematic review and meta-analysis of moderate-to-vigorous physical activity levels in secondary school physical education lessons
Source: Int J Behav Nutr Phys Act. 2017 Apr 24;14:52. doi: 10.1186/s12966-017-0504-0 (PMC5402678; doi:10.1186/s12966-017-0504-0)
Supplement: Additional file 1: — A record of the search strategy used for each database. (DOCX 24 kb) [file 12966_2017_504_MOESM1_ESM.docx]

**Additional File 1.**

**Database: MEDLINE**Name of Host: OVID
Number of results: 1128 (1058 after de-duplication)
Date searched: 20^th^ May 2014

| **#** | **Searches** | **Results** |
| --- | --- | --- |
| 1 | ((physical education or PE or physical activity or PA) adj5 (lesson* or class* or program* or curricul* or school* or instruct*)).mp. [mp=title, abstract, original title, name of substance word, subject heading word, keyword heading word, protocol supplementary concept word, rare disease supplementary concept word, unique identifier] | 5632 |
| 2 | (school adj3 sport*).mp. [mp=title, abstract, original title, name of substance word, subject heading word, keyword heading word, protocol supplementary concept word, rare disease supplementary concept word, unique identifier] | 606 |
| 3 | "Physical Education and Training"/ | 11777 |
| 4 | Exercise/ | 66233 |
| 5 | schools/ | 20707 |
| 6 | (3 or 4) and 5 | 1001 |
| 7 | 1 or 2 or 6 | 6628 |
| 8 | Adolescent/ | 1603007 |
| 9 | youth.mp. | 36545 |
| 10 | adolesc*.mp. | 1626365 |
| 11 | teenager*.mp. | 9881 |
| 12 | Child/ | 1354668 |
| 13 | ((primary or secondary or high or middle or elementary) adj5 (school* or student*)).mp. [mp=title, abstract, original title, name of substance word, subject heading word, keyword heading word, protocol supplementary concept word, rare disease supplementary concept word, unique identifier] | 44307 |
| 14 | 8 or 9 or 10 or 11 or 12 or 13 | 2262520 |
| 15 | Motor Activity/ | 77414 |
| 16 | physical activity.mp. | 50654 |
| 17 | Exercise/ | 66233 |
| 18 | fitness.mp. or Physical Fitness/ | 49372 |
| 19 | ((Moderate or vigorous) adj5 (physical activity or exercise)).mp. [mp=title, abstract, original title, name of substance word, subject heading word, keyword heading word, protocol supplementary concept word, rare disease supplementary concept word, unique identifier] | 9696 |
| 20 | MVPA.mp. | 994 |
| 21 | Movement/ | 59073 |
| 22 | 15 or 16 or 17 or 18 or 19 or 20 or 21 | 262721 |
| 23 | randomized controlled trial.pt. | 373289 |
| 24 | controlled clinical trial.pt. | 88322 |
| 25 | randomized.ab. | 271822 |
| 26 | randomised.ab. | 54401 |
| 27 | randomly.ab. | 193034 |
| 28 | trial.ab. | 281901 |
| 29 | groups.ab. | 1241003 |
| 30 | Cross-Sectional Studies/ | 174920 |
| 31 | prospective longitudinal quantitative studies.mp. | 0 |
| 32 | Prospective Studies/ | 365188 |
| 33 | Longitudinal Studies/ | 85628 |
| 34 | non randomized.ab. | 3820 |
| 35 | non randomised.ab. | 1748 |
| 36 | pre-post.mp. | 3932 |
| 37 | 23 or 24 or 25 or 26 or 27 or 28 or 29 or 30 or 31 or 32 or 33 or 34 or 35 or 36 | 2238313 |
| 38 | 7 and 14 and 22 and 37 | 1466 |
| 39 | limit 38 to yr="2005 -Current" | 1183 |
| 40 | limit 39 to english language | 1128 |

**Database: EMBASE**

Name of Host: OVID

Number of results: 2595 (1806 after de-duplication)

Date searched: 20^th^ May 2014

| **#** | **Searches** | **Results** |
| --- | --- | --- |
| 1 | ((physical education or PE or physical activity or PA) adj5 (lesson* or class* or program* or curricul* or school* or instruct*)).mp. [mp=title, abstract, subject headings, heading word, drug trade name, original title, device manufacturer, drug manufacturer, device trade name, keyword] | 13686 |
| 2 | (school adj3 sport*).mp. [mp=title, abstract, subject headings, heading word, drug trade name, original title, device manufacturer, drug manufacturer, device trade name, keyword] | 1825 |
| 3 | physical education/ | 9951 |
| 4 | exercise/ | 180665 |
| 5 | school/ | 42701 |
| 6 | (3 or 4) and 5 | 1623 |
| 7 | 1 or 2 or 6 | 15620 |
| 8 | adolescent/ | 1212474 |
| 9 | youth.mp. or juvenile/ | 54533 |
| 10 | adolesc*.mp. | 1277785 |
| 11 | teenager*.mp. | 13525 |
| 12 | child/ | 1271165 |
| 13 | ((primary or secondary or high or middle or elementary) adj5 (school* or student*)).mp. | 61723 |
| 14 | 8 or 9 or 10 or 11 or 12 or 13 | 2048397 |
| 15 | motor activity/ | 37340 |
| 16 | physical activity/ | 79300 |
| 17 | exercise/ | 180665 |
| 18 | fitness/ | 27860 |
| 19 | ((Moderate or vigorous) adj5 (physical activity or exercise)).mp. | 12597 |
| 20 | MVPA.mp. | 1326 |
| 21 | "movement (physiology)"/ | 26983 |
| 22 | 15 or 16 or 17 or 18 or 19 or 20 or 21 | 324876 |
| 23 | randomized controlled trial/ | 343796 |
| 24 | controlled clinical trial/ | 384560 |
| 25 | randomized.ab. | 381766 |
| 26 | randomised.ab. | 74822 |
| 27 | randomly.ab. | 261744 |
| 28 | trial.ab. | 391764 |
| 29 | groups.ab. | 1708919 |
| 30 | cross-sectional study/ | 112824 |
| 31 | prospective longitudinal quantitative studies.mp. | 0 |
| 32 | prospective study/ | 249489 |
| 33 | longitudinal study/ | 66210 |
| 34 | non randomized.ab. | 6672 |
| 35 | non randomised.ab. | 2444 |
| 36 | pre-post.mp. | 7135 |
| 37 | 23 or 24 or 25 or 26 or 27 or 28 or 29 or 30 or 31 or 32 or 33 or 34 or 35 or 36 | 2761230 |
| 38 | 7 and 14 and 22 and 37 | 3116 |
| 39 | limit 38 to yr="2005 -Current" | 2693 |
| 40 | limit 39 to english language | 2595 |

**Database: PsycINFO**

Name of Host: OVID

Number of results: 322 (120 after de-duplication)

Date searched: 20^th^ May 2014

| **#** | **Searches** | **Results** |
| --- | --- | --- |
| 1 | ((physical education or PE or physical activity or PA) adj5 (lesson* or class* or program* or curricul* or school* or instruct*)).mp. [mp=title, abstract, heading word, table of contents, key concepts, original title, tests & measures] | 4527 |
| 2 | (school adj3 sport*).mp. [mp=title, abstract, heading word, table of contents, key concepts, original title, tests & measures] | 580 |
| 3 | Physical Education/ | 3216 |
| 4 | Exercise/ | 14554 |
| 5 | exp Schools/ | 49334 |
| 6 | (3 or 4) and 5 | 337 |
| 7 | 1 or 2 or 6 | 5098 |
| 8 | Adolescent.mp. | 113619 |
| 9 | youth.mp. | 58604 |
| 10 | adolesc*.mp. | 194466 |
| 11 | teenager*.mp. | 6834 |
| 12 | Child.mp. | 256331 |
| 13 | ((primary or secondary or high or middle or elementary) adj5 (school* or student*)).mp. [mp=title, abstract, heading word, table of contents, key concepts, original title, tests & measures] | 164303 |
| 14 | 8 or 9 or 10 or 11 or 12 or 13 | 550695 |
| 15 | Motor Activity.mp. | 5483 |
| 16 | exp Physical Activity/ | 23610 |
| 17 | exp Exercise/ | 16598 |
| 18 | exp Physical Fitness/ or fitness.mp. | 11650 |
| 19 | ((Moderate or vigorous) adj5 (physical activity or exercise)).mp. [mp=title, abstract, heading word, table of contents, key concepts, original title, tests & measures] | 2247 |
| 20 | MVPA.mp. | 506 |
| 21 | Movement.mp. | 77278 |
| 22 | 15 or 16 or 17 or 18 or 19 or 20 or 21 | 114143 |
| 23 | randomized controlled trial*.mp. | 12477 |
| 24 | exp Clinical Trials/ | 7555 |
| 25 | randomized.ab. | 38271 |
| 26 | randomised.ab. | 4619 |
| 27 | randomly.ab. | 50644 |
| 28 | trial.ab. | 61644 |
| 29 | groups.ab. | 359321 |
| 30 | Cross-Sectional Stud*.mp. | 12396 |
| 31 | prospective longitudinal quantitative studies.mp. | 0 |
| 32 | exp Prospective Studies/ | 421 |
| 33 | exp Longitudinal Studies/ | 15241 |
| 34 | non randomized.ab. | 453 |
| 35 | non randomised.ab. | 147 |
| 36 | pre-post.mp. | 3703 |
| 37 | 23 or 24 or 25 or 26 or 27 or 28 or 29 or 30 or 31 or 32 or 33 or 34 or 35 or 36 | 485402 |
| 38 | 7 and 14 and 22 and 37 | 394 |
| 39 | limit 38 to yr="2005 -Current" | 326 |
| 40 | limit 39 to english language | 322 |

**Database: CINAHL**

Name of Host: EBSCO

Number of results: 632 (246 after de-duplication)

Date searched: 20^th^ May 2014

| # | Query | Results |
| --- | --- | --- |
| S38 | S7 AND S14 AND S22 AND S37 (English and 2005+) | 632 |
| S37 | S23 OR S24 OR S25 OR S26 OR S27 OR S28 OR S29 OR S30 OR S31 OR S32 OR S33 OR S34 OR S35 OR S36 | 579,673 |
| S36 | pre-post | 1,989 |
| S35 | AB non randomised | 684 |
| S34 | AB non randomized | 880 |
| S33 | "Longitudinal Studies" | 2,000 |
| S32 | (MH "Prospective Studies") | 232,563 |
| S31 | "prospective longitudinal quantitative stud*" | 0 |
| S30 | (MH "Cross Sectional Studies") | 88,432 |
| S29 | AB groups | 152,441 |
| S28 | AB trial | 52,874 |
| S27 | AB randomly | 32,891 |
| S26 | AB randomised | 14,040 |
| S25 | AB randomized | 53,314 |
| S24 | (MH "Clinical Trials") | 113,492 |
| S23 | (MH "Randomized Controlled Trials") | 31,119 |
| S22 | S15 OR S16 OR S17 OR S18 OR S19 OR S20 OR S21 | 74,744 |
| S21 | (MH "Movement") | 8,319 |
| S20 | MVPA | 368 |
| S19 | ((Moderate or vigorous) n5 (physical activity or exercise)) | 3,013 |
| S18 | (MH "Physical Fitness") OR "fitness" | 17,646 |
| S17 | (MH "Exercise") | 28,327 |
| S16 | (MH "Physical Activity") | 20,008 |
| S15 | (MH "Motor Activity") | 7,141 |
| S14 | S8 OR S9 OR S10 OR S11 OR S12 OR S13 | 471,711 |
| S13 | ((primary or secondary or high or middle or elementary) n5 (school* or student*)) | 27,256 |
| S12 | (MH "Child") | 276,395 |
| S11 | "teenager*" | 3,398 |
| S10 | adolesc * | 310,214 |
| S9 | "youth" | 17,555 |
| S8 | (MH "Adolescence") | 300,000 |
| S7 | S1 OR S2 OR S6 | 4,438 |
| S6 | (S3 or S4) and S5 | 551 |
| S5 | (MH "Schools") OR (MH "Schools, Elementary") OR (MH "Schools, Middle") OR (MH "Schools, Secondary") OR (MH "Schools, Nursery") | 13,762 |
| S4 | (MH "Exercise") | 28,327 |
| S3 | (MH "Physical Education and Training") | 1,769 |
| S2 | school n3 sport* | 328 |
| S1 | ((physical education or PE or physical activity or PA) n5 (lesson* or class* or program* or curricul* or school* or instruct*)) | 3,863 |

Database: **SPORTDISCUS**

Name of Host: EBSCO

Number of results: 1547 (1315 after de-duplication)

Date searched: 20^th^ May 2014

| # | Query | Results |
| --- | --- | --- |
| S38 | S7 AND S14 AND S22 AND S37 and English and 2005+ | 1,547 |
| S37 | S23 OR S24 OR S25 OR S26 OR S27 OR S28 OR S29 OR S30 OR S31 OR S32 OR S33 OR S34 OR S35 OR S36 | 143,126 |
| S36 | pre-post | 619 |
| S35 | AB non randomised | 93 |
| S34 | AB non randomized | 203 |
| S33 | Longitudinal Stud* | 4,217 |
| S32 | Prospective Stud* | 8,093 |
| S31 | prospective longitudinal quantitative studies | 0 |
| S30 | Cross-Sectional Stud* | 4,814 |
| S29 | AB groups | 101,653 |
| S28 | AB trial | 29,438 |
| S27 | AB randomly | 10,704 |
| S26 | AB randomised | 2,571 |
| S25 | AB randomized | 11,587 |
| S24 | Clinical Trial* | 10,209 |
| S23 | randomized controlled trial* | 7,029 |
| S22 | S15 OR S16 OR S17 OR S18 OR S19 OR S20 OR S21 | 328,852 |
| S21 | Movement | 49,594 |
| S20 | MVPA | 519 |
| S19 | ((Moderate or vigorous) n5 (physical activity or exercise)) | 4,479 |
| S18 | fitness | 148,806 |
| S17 | Exercise | 190,080 |
| S16 | Physical Activity | 44,363 |
| S15 | Motor Activity | 1,988 |
| S14 | S8 OR S9 OR S10 OR S11 OR S12 OR S13 | 158,680 |
| S13 | ((primary or secondary or high or middle or elementary) n5 (school* or student*)) | 36,787 |
| S12 | Child | 90,756 |
| S11 | teenager* | 34,903 |
| S10 | adolesc* | 27,005 |
| S9 | youth | 23,120 |
| S8 | Adolescence | 8,127 |
| S7 | S1 OR S2 OR S6 | 41,453 |
| S6 | (S3 or S4) and S5 | 33,194 |
| S5 | Schools | 148,418 |
| S4 | Exercise | 190,080 |
| S3 | Physical Education and Training | 55,166 |
| S2 | school n3 sport* | 14,522 |
| S1 | ((physical education or PE or physical activity or PA) n5 (lesson* or class* or program* or curricul* or school* or instruct*)) | 1 |

**Database: Cochrane Database of Systematic Reviews**

Name of Host: Wiley

Number of results: 6 (3 after de-duplication)

Date searched: 20^th^ May 2014

'(("physical education" OR PE OR "physical activity" OR PA) NEAR/5 (lesson* OR class* OR program* OR curricul* OR school* OR instruct*)) OR (school NEAR/3 sport*) OR (exercise NEAR/5 school*) in Title, Abstract, Keywords and Adolesce* OR teenager* OR youth OR child OR ((primary OR secondary OR high OR middle OR elementary) NEAR/5 (school* OR student*)) in Title, Abstract, Keywords and "Motor Activity" OR "physical activity" OR exercise OR fitness OR ((Moderate OR vigorous) NEAR/5 ("physical activity" OR exercise)) in Title, Abstract, Keywords in Cochrane Reviews'

**Database: CENTRAL (Cochrane Central register of Controlled Trials)**

Name of Host: Wiley

Number of results: 410 (62 after de-duplication)

Date searched: 20^th^ May 2014

**Cochrane Central Register of Controlled Trials : Issue 4 of 12, April 2014**

## There are 410 results from 789657 records for your search on '(("physical education" OR PE OR "physical activity" OR PA) NEAR/5 (lesson* OR class* OR program* OR curricul* OR school* OR instruct*)) OR (school NEAR/3 sport*) OR (exercise NEAR/5 school*) in Title, Abstract, Keywords and Adolesce* OR teenager* OR youth OR child OR ((primary OR secondary OR high OR middle OR elementary) NEAR/5 (school* OR student*)) in Title, Abstract, Keywords and "Motor Activity" OR "physical activity" OR exercise OR fitness OR ((Moderate OR vigorous) NEAR/5 ("physical activity" OR exercise)) in Title, Abstract, Keywords in Trials'

**Database: ERIC**

Name of Host: Proquest

Number of results: 192 (81 after de-duplication)

Date searched: 20^th^ May 2014

((“physical education” OR PE OR “physical activity” OR PA) NEAR/5 (lesson* OR class* OR program* OR curricul* OR school* OR instruct*)) OR (school NEAR/3 sport*) OR (exercise NEAR/5 school*)

AND

Adolesce* OR teenager* OR youth OR child OR ((primary OR secondary OR high OR middle OR elementary) NEAR/5 (school* OR student*))

And

“Motor Activity” OR “physical activity” OR exercise OR fitness OR ((Moderate OR vigorous) NEAR/5 (“physical activity” OR exercise))

AND

(“randomized controlled trial*” OR “clinical trial*” OR randomized OR randomised OR randomly OR trial OR groups OR “Cross-Sectional Stud*” OR “prospective longitudinal quantitative stud*” OR “Prospective Stud*” OR “Longitudinal Stud*” OR “non randomized” OR “non randomised”)

**Database: SCOPUS**

Name of Host: SCOPUS

Number of results: 1468 (517 after de-duplication)

Date searched: 20^th^ May 2014

((“physical education” OR PE OR “physical activity” OR PA) W/5 (lesson* OR class* OR program* OR curricul* OR school* OR instruct*)) OR (school sport*)

AND

Adolesce* OR teenager* OR youth OR child OR ((primary OR secondary OR high OR middle OR elementary) W/5 (school* OR student*))

And

“Motor Activity” OR “physical activity” OR exercise OR fitness OR ((Moderate OR vigorous) W/5 (“physical activity” OR exercise))

AND

(“randomized controlled trial*” OR “clinical trial*” OR randomized OR randomised OR randomly OR trial OR groups OR “Cross-Sectional Stud*” OR “prospective longitudinal quantitative stud*” OR “Prospective Stud*” OR “Longitudinal Stud*” OR “non randomized” OR “non randomised”)
