# Supplementary material for: A systematic review and meta-analysis of moderate-to-vigorous physical activity levels in secondary school physical education lessons
Source: Int J Behav Nutr Phys Act. 2017 Apr 24;14:52. doi: 10.1186/s12966-017-0504-0 (PMC5402678; doi:10.1186/s12966-017-0504-0)
Supplement: Additional file 2: — 11-item risk of bias tool developed for the systematic review. (DOCX 14 kb) [file 12966_2017_504_MOESM2_ESM.docx]

**Additional File 2: Risk of bias assessment for PE lesson MVPA systematic review**

**Study number: Authors:**

**Review type: Year of publication:**

| **Risk of bias criteria** | **Answer** |
| --- | --- |
| **School level** |  |
| 1. **Did the study adequately describe the key demographic characteristics of the school sample? i.e. SES and/or geographical location.**   Y = yes, the study adequately described the school characteristics including SES and/or geographical location; N = no, the study did not adequately describe the school characteristics; U = unclear. |  |
| 1. **Was the school sample representative of the population? i.e. schools randomly selected from region.**   Y = yes, the school/s were randomly selected from the population or all schools from a region were invited to participate; N = no, the school/s were not randomly selected e.g. convenience sampling or if stated they were not representative; U = unclear. |  |
| **Class level** |  |
| 1. **Was the class chosen representative of all school classes? i.e. class of students randomly selected or an entire grade/s invited to participate.** Y = yes, the class/es were randomly selected or all classes from a grade/s were invited to participate; N = no, the class/es were not randomly selected e.g. convenience sampling; U = unclear. |  |
| 1. **Did the study adequately describe the key demographic characteristics of the class sample? i.e. grade, sex breakdown.**   Y = yes, the study adequately described the class characteristics (i.e. grade, sex breakdown); N = no, the study did not adequately describe the class characteristics; U = unclear. |  |
| **Student level** |  |
| 1. **Did the study adequately describe the participant eligibility criteria? i.e. grade, age.**   Y = yes, the study adequately described the participant eligibility; N = no, the study did not adequately describe the participant eligibility criteria; U = unclear. |  |
| 1. **Did the study adequately describe the key demographic characteristics of the student sample? i.e. number of participants and their mean age (or age range) and sex breakdown.**   Y = yes, the study adequately described the number of students who participated, mean age (or age range) and sex; N = no, the study did not adequately describe the number of participants, mean age (or age range) and/or sex; U = unclear. |  |
| 1. **Was the student sample representative of the population? i.e. students measured were randomly selected or an entire grade/s invited to participate/measured.** Y = yes, the students were randomly selected from the population or all participants from a grade/s invited to participate/measured; N = no, the students were not randomly selected e.g. convenience sampling; U = unclear. |  |
| **PE lesson observation** |  |
| 1. **Did the study adequately describe the number of PE lessons observed?**   Y = yes, the study adequately described the number of PE lessons observed; N = no, the study did not adequately describe the number of PE lessons observed; U = unclear. |  |
| 1. **Did the study use an objective measure of physical activity (i.e. accelerometers, heart rate monitors, pedometers) or did the study cite validation studies or state validity data for observational measures in the study population (e.g. primary and secondary school children).** Y = yes, the study used an objective measure of physical activity, or used an objective measure in a sub-sample of students, or used observational measures and cited validation studies/stated validity data in the study population being examined; N = no, the study did not used objective measures, or did not cite a validation study/validation data in the population being studied; U = unclear. |  |
| 1. **Did the study use an objective measure of physical activity (i.e. accelerometers, heart rate monitors, pedometers) or did the study state reliability data or cite reliability studies for observational measures in the study population (e.g. primary and secondary school children).** Y = yes, the study used an objective measure of physical activity, or used an objective measure in a sub-sample of students, or used observational measures and cited reliability studies/stated reliability data in the study population being examined (inter-rater reliability: ICC > .70 is considered acceptable); N = no, the study did not used objective measures, or did not cite reliability study/data in the population being studied; U = unclear. |  |
| 1. **Did the study report the nature of the physical activities observed?**   Y = yes, the study reported the type of activities observed (e.g. type of sport or game); N = no, the study did not reported the type of activities observed; U = unclear. |  |
